# Central and Peripheral Fatigue During Resistance Exercise – A Critical Review

**DOI:** 10.1515/hukin-2015-0118

**Published:** 2015-12-30

**Authors:** Adam Zając, Małgorzata Chalimoniuk, Adam Maszczyk, Artur Gołaś, Józef Lngfort

**Affiliations:** 1 Department of Sports Theory, The Jerzy Kukuczka Academy of Physical Education, Katowice, Poland; 2 The Department of Tourism and Health in Biała Podlaska, Józef Piłsudski University of Physical Education in Warsaw, Poland

**Keywords:** resistance exercise, muscle contraction, metabolites, excitability, fatigue

## Abstract

Resistance exercise is a popular form of conditioning for numerous sport disciplines, and recently different modes of strength training are being evaluated for health benefits. Resistance exercise differs significantly in nature, and several variables determine the direction and range of adaptive changes that occur in the muscular and skeletal system of the body. Some modes of resistance training can also be effective in stimulating the cardiovascular system. These variables include exercise selection (general, specific, single or multi joint, dynamic, explosive), type of resistance (free weights, variable resistance, isokinetics), order of exercise (upper and lower body or push and pull exercises), and most of all the training load which includes intensity expressed as % of 1RM, number of repetitions, number of sets and the rest interval between sets. Manipulating these variables allows for specific adaptive changes which may include gains in muscle mass, muscle strength or muscle endurance. It has been well established that during resistance exercise fatigue occurs, regardless of the volume and intensity of work applied. The peripheral mechanisms of fatigue have been studied and explained in more detail than those related to the CNS. This review is an attempt to bring together the latest knowledge regarding fatigue, both peripheral and central, during resistance exercise. The authors of this review concentrated on physiological and biochemical mechanisms underlying fatigue in exercises performed with maximal intensity, as well as those performed to exhaustion with numerous repetitions and submaximal load.

## Introduction

Resistance exercise that requires both shortening (concentric) and lengthening (eccentric) contractions is a mode of training commonly accepted as an integral part in the athletes training regimen. The major objective of resistance exercise is the stimulation of structural and functional adaptation in the organism to improve performance in a sport specific physical task and/or induce health-related benefits. In case of athletes, to achieve this target, a carefully planned training program must be applied that focuses on appropriate frequency, length (volume) and intensity of work, and includes optimal rest intervals. Effectiveness of mechanical stimuli on muscle tissue also depends on type of contractions i.e. lengthening contractions have shown a more powerful stimulus for neuromuscular adaptation compared to shortening ones (Adams et al. 2004, Hortobagyi et al. 1996, Zebrowska et al. 2013b). Application of these factors varies depending on a given individual’s state of performance and fitness. It is well established that an efficient training program should be based on the so called overload principle which is defined as the workload demand on the body greater than that to which it is accustomed (Bompa 2005). This principle commonly accomplished by coaches inevitably leads to frequently experienced by athletes, acute post exercise fatigue (Fry et al. 1991, Borresen and Lambert, 2009, Bangsbo et al. 2013). Adaptive changes in response to exercise occur only during rest, thus improper post exercise recovery may cause residual fatigue (Borresen and Lambert, 2009), and as a consequence the athlete may not reach the supercompensation phase (Zatsiorsky and Kraemer 2006). Systemic residual fatigue carries on to overtraining, defined as a state of overstress or failure to adapt to an exercise load and/or to a drop in performance level. In extreme cases, when not compensated it leads to the overtraining syndrome (Johnson and Thiese 1992, Kreher and Schwartz 2012). The overtraining syndrome is expressed in the inability to train hard, and occurs in two clinical forms: sympathetic and parasympathetic and generally their appearance are strongly depended on the type and mode of exercise applied. Both forms of fatigue may involve central, e.g. CNS, (Gandevia 2001) and/or peripheral sites (Enika and Duchateau 2008). Studies indicate that peripheral fatigue appears when depletion of energy stores occurs, accumulation of by-products or impairment of muscle contractile mechanism is attained in response to exercise, and recently its relation to an immunological and genetic response is suggested (Poole et al. 2008, Jones et al. 2008, Vanhatalo et al. 2010, Keyser 2010, Finsterer 2012). Changes in performance in relation to the above mentioned factors were carefully investigated in humans in response to different types of exercise, yet they can’t fully explain fatigue symptoms appearing during endurance exercise (Bigland-Richte et al. 1986, Cooper et al. 1988, Noakes, 2012). Thus, declines in performance during exercise are also attributed to CNS, which integrates input from various body parts and is known as a central fatigue. In case of resistance training, central fatigue is poorly investigated and recognized.

## Resistance exercise and local fatigue

In general, desired adaptation in response to strength training can be induced by specific type of loading. Mostly two types of loading in strength training protocols are used, e.g. hypertrophic loading, and the so called maximal strength training. In both cases the amount of work performed, and especially the intensity of work with a proper recovery phase play a crucial role in generating muscle growth (Werborn et al. 2007). Evidence presented in the literature highlights that both types of loading cause similar post-exercise intramuscular perturbations, e.g. decrease in intramuscular glycogen, phosphocreatine, ATP stores and an increase in inorganic phosphate and hydrogen ions (Dawson et.al. 1978, Hivonnen et al. 1987, Green 1997, Westerblad et al. 2002, Takada et al. 2012). These changes are especially evident when exercise intensity during training sessions reach or are close to one repetition maximum (1RM) (Tesch eta al. 1986). This leads to muscle fatigue, and in turn to a reduction of skeletal muscle function. There is now evidence for the existence of a common physiological mechanism that may be involved in eliciting muscle fatigue and pain (Burnes at al. 2008). Nociceptive afferent input may be involved in the modulation of central motor drive during exercise, and impaired motor function to prevent the development of fatigue (Amann et al. 2009). There are a few well recognized activating factors at the cellular level for group III and IV muscle afferents: ATP, inorganic phosphate and H+ ions (Allen et al. 2008). Release of ATP usually occurs in patients with muscle damage due to trauma (Mense 2008). It is known that resistance exercise also induces muscle damage as indicated by muscle micro injures (Ribeiro 2008), and increased serum CK activity (Nosaka and Clarkson 1992, Rodriguez 2010, Jackman et al. 2010). Elevated plasma CK activity has been proposed as one of the best indirect indicator of muscle damage, especially in the case of resistance exercise (Koch et al. 2014). Some studies indicate an association between serum CK level, and both strength impairment and reduced ATP production after resistance exercise (Brancaccio et al. 2007, Brancaccio et al. 2010, Sorichter et al. 1999). This type of exercise habitually performed in ischemic conditions simultaneously causes a fall in pH (Webster et al. 1993, Westerblad et al. 2002). Metabolic and chemical perturbations in skeletal muscle were also linked with transient fatigue after intermittent-sprint exercise performed with maximal intensity (Bangsbo et al. 2006, Kayser 2010). Evidence presented in human and animal studies highlight the connection between changes in metabolic products of muscular contraction and CNS function, by stimulation of III and IV muscle afferents. Myelinated group III afferents are more mechanosensitive, and are stimulated preferentially in response to muscle force production (Rotto and Kaufman 1988, Hayes et al. 2009). Unmyelinated group IV afferents are primarily metabosensitive, as compared to group III (Haouzi et al. 1999). In regards to recent animal studies two different subtype of chemosensitive group III and IV muscle afferents were distinguished: metaboreceptors (ergoreceptors) and metabo-nociceptors, but its mutual participation in stimulating supraspinal areas is not known (Jankowski et al. 2013, Amann et al. 2015). During muscle contractions both groups of afferents transmit information to the brainstem to adjust cardiorespiratory activity by increased sympathetic nerve activity (Murphy at al. 2011). It is not known how this mechanism operates during resistance exercise, despite the fact that dramatic increases in both blood pressure (BP) and heart rate in response to this type of exercise have been observed for years (Niewiadomski et al. 2008, Sale et al. 1993, Scharf et al. 1994, Zebrowska et al. 2013a). The rise in BP may be at least partially explained by compression of the vasculature from intracellular forces generated by muscles during static exercise (Lydakis et al. 2008). Moreover, it was shown that during exhaustive contraction activation of group III and IV muscle afferents affect the regulation of motor unit recruitment by operating at spinal and supraspinal levels (Gandevia 2001, Decherchi and Dousset 2003), and participate in motor command adjustment (Amman and Dempsey 2008, Wang et al. 2010). It is not fully recognized which of these nerve fibers are mainly activated during resistance exercise. With regard to exhaustive contraction, activation of muscle afferents should especially occur during resistance exercise performed with intensity close to 1RM to prevent the magnitude of muscle fatigue and the danger of injuries (Taylor et al. 2000). It is known that recruitment of muscle fiber types depend on the intensity of exercise performed, and type I fibers are recruited when exercise starts, whereas type II fibers are activated when exercise intensity increases (Merletti et al. 1990). The consequences of such a recruitment pattern during exercise performed with intensity close to 1RM is that recruitment of both the type I and II motor units occurs. Because type I muscle fibers are O2–dependent, resistance exercise performed with intensity close to 1RM may cause a transient local hypoxic condition. This may suggest that peripherally-induced factors in response to resistance exercise should be primarily involved in activation of type III/IV muscle afferents within type I muscle fibers. In line with such an assumption, type I muscle fibers are always initially recruited during exercise (Sale 1987). On the other hand type II fibers achieve recruitment during high intensity fatiguing exercise and therefore it is suggested that a high share of II muscle fibers are recruited to perform resistance exercise with intensity close to 1RM (Komi and Tesch 1979). Following this notion, during brief maximal exercise, a greater metabolic stress indicated by reduction in ATP was demonstrated in human type II fibers in comparison to I type (Karatzaferi at al. 2001). Similarly, the decrease of ATP content during recovery was still lower in type II fibers than type I (Karatzaferi et al. 2001a). It is clear that an increase in lactate production mirrors recruitment and activity of type II muscle fibers, and this process starts at exercise intensity reaching the anaerobic threshold (Peinado et al. 2014). Accordingly, a high level of blood lactate levels was seen, after both unilateral and bilateral resistance exercise in young man with at least 6 month strength training experience (Costa et al. 2015). Thus, a sudden increased acidosis in type II fibers, and a high rate of phosphocreatine hydrolysis accompanied by concomitant inorganic phosphate accumulation during resistance exercise may be considered as a predominant factor stimulating type III/IV afferents. Some authors indicate that peripheral fatigue manifesting itself as a muscle locomotor impairment is very sensitive for hypoxemia and hypoxia conditions (Romer et al. 2006, Romer et al. 2007). This leads to the assumption that diminished oxygen delivery during resistance exercise, which has a considerable isometric component is a substantial factor affecting intramuscular metabolic perturbations. However, a recent study concerning handgrip exercise with blood flow occlusion provides evidence that the magnitude of fatigue development is not constant for small muscle mass exercise across O2 delivery conditions (Broxterman et al. 2015). In addition to this, recent studies with the utilization of nuclear magnetic resonance showed that blood flow restricted and traditional resistance training performed to fatigue produced equal muscle hypertrophy (Farup et al. 2015). Currently, it is not fully recognized which of group III and IV muscle afferents are mainly activated during resistance exercise and this issue needs to be further investigated. A role of group III and IV muscle afferents on central motor drive has been lately reviewed by Laurin et al. (2015).

## Central fatigue and resistance exercise

Limitations to strength are both neuromuscular and muscular in nature (Scale 1998). Participation of CNS in strength generation is indirectly demonstrated by adaptation to resistance training seen without increasing in muscle girth during the initial weeks of strength training (McArdle et al. 2009). Research indicates that both central and peripheral fatigue expand more slowly during submaximal exercises in comparison to maximal voluntary contractions (Taylor and Gandevia 2008). Among potential mediators of CNS related fatigue, depletion of acetylcholine in the motor end plate may play a negligible role because neuronal impulses are not blocked during muscle fatigue at the neuromuscular junction structure, and acetylcholine is not fully depleted (Fitts 1994, Baker et al. 1993). This leads to the conclusion that motor neuron drive during resistance exercise must be regulated by higher structures in the central nervous system, and feedback signals from the muscle. The locomotor system is organized by neuronal networks of central pattern generators (CPGs) located in the spinal cord responsible for producing the basic rhythmic patterns (Guertin 2012). Higher-level centers (the prefrontal cortex, the motor cortex, basal ganglia and cerebellum) are responsible for the initiation of movement and modulation of its patterns to environmental conditions by changing its speed and direction (Grillner, 1997). The importance of all these brain regions for the generation of voluntary rhythmic motor patterns was documented by functional magnetic resonance imaging in humans (Jahn et al. 2004, 2008). Locomotor movements may also be shaped by nitric oxide (NO) acting as a meta-modulator by affecting other neuromodulators / neurotransmitters of monoaminergic brain system (McLean and Sillar, 2004, Chalimoniuk et al. 2005, Goekint et al. 2012), and recently the alternations in the nitric oxide/soluble guanylyl cyclase/cyclic guanosine 3′,5′-monophosphate pathway in motor control-related subcortical brain regions were suggested to take part in regulation of locomotor activity after endurance training (Chalimoniuk et al. 2015). In agreement with a possible involvement of resistance exercise in modulation of NO-dependent pathways are data showing participation of NO/cGMP/KATP pathway in ant nociception in rats. In this study increasing nitrite levels was seen both in plasma and CNS in resistance exercised rats (Galdino et al. 2015). Among CNS monoamines especially serotonin (5-HT), dopamine (DA) and noradrenaline (NA) have been merged with central fatigue by investigating micro dialysis probe from brain areas involved in control of locomotion (Meeusen 2005, Meeusen et al. 2007). Changes in 5-HT, as proposed by Newsholme et al. (1991) modulates neurotransmission and numerous physiological functions leading to central fatigue and attenuating exercise performance (Meeusen et al. 2006). This hypothesis assumes that elevation of plasma free fatty acids (FFA) and/or increase in muscle uptake and metabolism of branched-chain amino acids (BCAA) during exercise augments the ratio of unbound tryptophan. The increased amount of tryptophan facilitates its crossing through the blood-brain barrier, and therefore leading to higher 5-HT concentration within the brain potentially affecting performance. Referring to this hypothesis drawn out from the data of exhausting endurance exercise to resistance exercise, it’s worth indicating, that this type of exercise does not significantly affect plasma FFA concentration (Goto et al. 2007, Rezaee et al. 2014), but may increase the rate of BCAA metabolism, and thus reducing its plasma concentration (Shimomura et al. 2009). Therefore, it can be postulated that during resistance exercise a shift in plasma BCAA concentration may be an important factor affecting central fatigue. On the other hand, nutritional strategy with BCAA supplementation have been shown to improve muscle repair after resistance exercise-induced muscle damage (Shimomura et al. 2009, da Luz et al. 2011), and considerably reduce muscle soreness (Jackman et al. 2010). Therefore, it can be speculated that BCAA-induced reduction in muscle damage after resistance exercise may attenuate the involvement of group III/IV afferents in the development of central fatigue. However, their inhibitory effect on the output from spinal motoneurons (Amman et al. 2008, Amman et al. 2009) during resistance exercise needs to be investigated. It seems likely that a possible BCAA protective effect against muscle damage differs in the so called high and normal responders (Machado and Willardson 2010). One can speculate that it can happen at the skeletal microRNA level because high responders demonstrate different regulation of selected miRNA expression in comparison to low responders (Davidsen et al. 2011). The tryptophan-serotonin theory has been expanded from data obtained in animal studies by indication that 5-HT release and reuptake can affect central fatigue (Bequet et al. 2002). Moreover, some authors introduced the concept of the ratio of 5-HT to DA, and other neurotransmitters as more closely related variables with central fatigue (Meeusen et al. 2006, Meeusen and Watson 2007). Upregulation of DA in the brain has been linked to exercise-induced elevation of blood calcium and its enlarged influx into CNS (Sutoo and Akiyama 2003). The responsible mechanisms for a rise in brain DA after endurance training has been attributed to both calcium/calmodulin-dependent DA synthesis and increased DA receptor affinity to DA (McRae et al. 1987, Sutto and Akiyama 2003). In this regard, there is a lack of cross sectional studies focused on resistance exercise. In spite of all, plasma level of calcium after resistance exercise was shown to reveal a biphasic pattern; lowering of calcium occurred during the first two hours after cessation of resistance exercise, and a significant elevation from the second hour was recorded for a further two hours in sedentary subjects (Ashinzawa et al. 1997). In this study, during the entire examined period after cessation of resistance exercise, hypercalciuria appeared accompanied by acidosis (Ashinzawa et al. 1997), and the latter is known to stimulate the efflux of calcium from the bone (Bushinsky et al. 1983, Gren and Kleeman 1991). Hydroxylation of DA leads to NA synthesis. The noradrenergic neurons innervate the whole cerebral cortex, different subcortical areas, cerebellum and the brain stem (Bouret et al. 2005), with all of them involved in the regulation of movement. In case of NA, there are studies which attempted to investigate memory; learning and cognitive function during exercise and elevation of NA within the CNS was shown to improve the aforementioned functions (Dunn et al. 1996, Sarbadadhikari and Saha 2006, Ebrahimi et al. 2010). A significant role of DA and NA in control of performance was also documented during exercise in the heat (Meeusen et al. 2006). There is a lack of direct studies on central fatigue in response to resistance exercise. However, a recent study performed by means of Heart Rate Variability analysis indirectly indicates a pronounced activation of the autonomic nervous system after high-intensity body weight resistance exercise (Brian 2015).

Research investigating brain activity related to central fatigue shows that neurotransmission can be influenced by byproducts of muscle metabolism and the effect of ammonia has been mostly explored. Under normal physiological conditions ammonia predominately appears in blood as the metabolic end product from gut and in a smaller extent by the gastrointestinal tract (Romero-Gomez et al. 2009). Skeletal muscle has got a large capacity for ammonia production, what is usually revealed by its high blood accumulation during exercise above 60 VO2max (Buono et al. 1984). Diet manipulation is another potent factor that may increase ammonia production during exercise of equal intensity, and it can entail dissociation between lactate and ammonia thresholds in healthy young men, commonly applied indicators for control of the training process (Czarnowski et al. 1995, Langfort et al. 2004). Rapid adenosine tri-phosphate hydrolysis during high intensity exercise builds up adenosine di-phosphate and adenosine monophosphate (AMP). In the further metabolic degeneration cascade known as the purine nucleotide cycle, AMP is deaminated into inosinomono-phosphate, with the parallel formation of ammonia (NH3). Because ammonia is correlated with the number of fast switch muscle fibers, an increase in lactate and efficacy of oxidative metabolism (Meyer et al. 1980, Jansson et al. 1987, Dudley and Terjung 1985, Wilkinson et al. 2010), and all these factors are linked with resistance exercise, this may suggest that ammonia might be an important player in modulation of central fatigue during resistance exercise. Such an effect was previously seen during prolonged exercise in humans and it was postulated that ammonia provoked central fatigue by affecting neurotransmitter metabolism (Nybol et al. 2005). There is a consensus that the impact of hiperammonemia on brain function can be seen when elevated systemic ammonia level sets up to 200 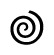
mol × l-1 (Banister and Cameron 1990, Brouns et al. 1990, Nybo et al. 2005, Mohr et al. 2006). However, there is no research in this area in case of resistance exercise. The available data from prolonged endurance exercise and patients with liver disease may lead to the conclusion that induced in response to intense resistance exercise hiperammonemia does not have deteriorations in brain function. This is because the first symptoms of deterioration of CNS functions emerge with a delay of a few hours (~2 hrs) after eliciting hiperammonemia. Secondly, a higher uptake of ammonia by CNS may be “buffered” by the protective role of astrocytes. The above mentioned issues and ammonia metabolism in relation to brain and fatigue have been excellently reviewed and discussed by Wilkinson et al. (2010) despite the fact that the authors did not pay any attention to sweat ammonia excretion during exercise (Czarnowski and Górski 1991, Czarnowski et al. 1992).

## Conclusions and perspectives

Recent human and animal experiments indicate that peripheral and central fatigue develops more slowly during submaximal exercise compared to maximal voluntary contractions (Taylor and Gandevia 2008). In this context, mechanisms underlying fatigue during high intensity resistance exercises have been poorly investigated. This fully justifies the designing of well controlled resistance exercise studies to reveal possible mechanisms of central fatigue using hypertrophy, maximal strength and power modes of exercise with both highly trained athletes and untrained subjects, to additionally determine adaptive mechanisms. Additional experiments can be carried out with the use of state-of the art technology, including magnetic resonance imaging and spectroscopy as well as positron-emission-tomography. The above mentioned techniques should be utilized with supplements preventing or delaying central fatigue and those inducing it during high intensity resistance exercise. Another possible approach to explain mechanisms responsible for central fatigue induced by resistance exercise could be revealed in studies with pharmacological manipulation of monoamine receptors. At the cellular level, a role in peripheral fatigue of some metabolites as prostaglandins, thromboxane, bradykinin and purinergic type 2X receptors (e.g. P2X and TPRv1) as well as ion channels (e.g. ASICs) in response to resistance exercise also needs to be investigated.
